# Action Planning Checklist for Social Determinants of Health: Older Adults with Chronic Conditions

**DOI:** 10.1093/geroni/igab046.3350

**Published:** 2021-12-17

**Authors:** Joan Ilardo, Angela Zell

**Affiliations:** Michigan State University College of Human Medicine, East Lansing, Michigan, United States

## Abstract

Medical residents need training to assess social determinants of health (SDOH) related to chronic conditions. We created a checklist to identify SDOH affecting residency clinic patients’ ability to manage chronic conditions. The tool: 1) involves resident training; 2) provides decision support checklist; 3) influences patient activation; and 4) increases provider and patient communication through shared decision making. Action Planning Guide checklist (APG) includes questions pertaining to SDOH preventing patients from managing their chronic conditions and actions patients will take. Areas identified are discussed between patient and resident, increasing patient activation. The clinic’s nurse care facilitator guides referrals to community-based resources. Fifty-two patients were enrolled, with 75% of patients responding they would like to be better managers of their chronic conditions. This information is used to develop patient’s goals of care. Over 90% of patients said their conditions affect their lives and discussed ways better to care for themselves. Over 80% discussed medication management, health goals to improve their quality of life, and made a plan that maps out ways to reach their goals. All of these are essential for achieving positive health outcomes for older patients with chronic conditions. These attributes promote effective patient/provider partnerships. Seventy referrals were made; food through 2-1-1 (47%); monthly commodity food program (30%); utility payments (11%), and transportation (9%). Twenty-seven referrals were made to agencies serving older adults; 25 to the local AAA information and assistance services, and 2 to Senior Project Fresh Voucher Program.

